# Supplementary material for: Content-rich biological network constructed by mining PubMed abstracts
Source: BMC Bioinformatics. 2004 Oct 8;5:147. doi: 10.1186/1471-2105-5-147 (PMC528731; doi:10.1186/1471-2105-5-147)
Supplement: Additional File 2 — The original results of the above study (non-essential files are deleted to keep the file size under the limit set by BMC bioinformatics). [file 1471-2105-5-147-S2.bz2 › chilibotAdditionalFile2/dip50/271ID2169352E1091/html/NR3C1_FOS.html]

 


 **NR3C1** and **FOS** 
  
Found 90 abstracts in PubMed, retrieved 50.  
 

 What does Google say? 
 PDF only 
| .edu only 

---

**Interactive relationship** (e.g. stimulation, inhibition, etc)

**Inhibitory relationship**- Importantly, AP 1 responsiveness for glucocorticoid receptor  [ **NR3C1** ]  mediated inhibition was preserved in  **Fos**  Fosl1 Fosl1 mice.  Ref: 11953459 J Neurochem, 2002
- It has recently been reported that the glucocorticoid receptor  [ **NR3C1** ]  inhibits activity of the AP 1 transcription factor by the ligand dependant binding of glucocorticoid receptor  [ **NR3C1** ]  GR to the  **fos**  and jun components of AP 1.  Ref: 10500841 Adv Exp Med Biol, 1999
- Transgenic TG mice expressing an antisense mRNA against the glucocorticoid receptor  [ **NR3C1** ]  GR, which partially blocks GR expression, were used to assess the role of GR dysfunction on cocaine COC induced c  **fos**  and Nerve Growth Factor Inducible B NGFI B, or Nur77 gene expression.  Ref: 12629527 Neuropsychopharmacology, 2003
**Neutral relationship**- No group differences were found in plasma corticosterone levels or mRNA levels of glucocorticoid receptor  [ **NR3C1** ] , corticotropin releasing hormone, 5 HT 2C or c  **fos** .In conclusion, we have found that social interaction is a major component of the enriched environment regarding the effects on NGFI A and NGFI B expression.  Ref: 12809685 Neuroscience, 2003

**Non-interactive relationship** (e.g. studied together, co-existance, homology, etc.)

- This impairment may result from the absence of c  **Fos**  or glucocorticoid receptor  [ **NR3C1** ]  mediated transrepression.  Ref: 12766089 Invest Ophthalmol Vis Sci, 2003
- Our studies showed that the expressions of the genes including glucocorticoid receptor  [ **NR3C1** ]  GR, mineralocorticoid receptor MR, bcl 2, c  **fos** , neural cell adhesion molecule NCAM, presenilin 2 PS 2 and apoE in the hippocampus were closely related to the central learning and memory function in senescence accelerated mice SAM, hydrocortisone HC treated mice and normal mice.  Ref: 12545709 Sheng Li Ke Xue Jin Zhan, 2000
- Following an isotonic saline injection the time course of TH, c  **fos**  and glucocorticoid receptor  [ **NR3C1** ]  mRNA was examined.  Ref: 11287061 Brain Res Dev Brain Res, 2001
- This effect may be due to a functional antagonism, at the transcriptional level, between  **Fos**  and the glucocorticoid receptor  [ **NR3C1** ] .  Ref: 8681202 Rev Neurol, 1996
- Expression of c  **fos** , rather than c jun or glucocorticoid receptor  [ **NR3C1** ]  mRNA, correlates with decreased glucocorticoid response of peripheral blood mononuclear cells in asthma.  Ref: 12400872 Int Immunopharmacol, 2002
- Influence of the glucocorticoid receptor  [ **NR3C1** ]  on c  **fos**  inducibility in activated ras containing mouse lung cells.  Ref: 8890955 Mol Carcinog, 1996
- These data demonstrate that CRF is a potent stimulus for corticotroph expression of c  **fos** , jun B and  **fos**  B, and suggest that the subsequent increase in AP 1 may play a role in activation of gene expression and or as a modulator of glucocorticoid receptor  [ **NR3C1** ]  function.  Ref: 8793851 Mol Cell Endocrinol, 1996
- The glucocorticoid receptor  [ **NR3C1** ]  GR is a ligand activated nuclear transcription factor, and AP 1  **Fos**  Jun or Jun Jun is a transcription factor whose components are nuclear proteins encoded by c  **fos**  and c jun protooncogenes.  Ref: 9657066 Endocrine, 1997
- The deprived pups also showed in the paraventricular nucleus a marked increase of stress induced c  **fos**  mRNA and a reduction of corticotropin releasing hormone and glucocorticoid receptor  [ **NR3C1** ]  mRNA.  Ref: 9822770 J Neurosci, 1998
- ... c myc, c jun, c  **fos** , and glucocorticoid receptor  [ **NR3C1** ]  mRNAs were downregulated by 23 43%.  Ref: 10329965 Am J Physiol, 1999
- The levels of Alzheimer s disease related genes, including beta amyloid precursor protein, presenilin 1, PS 2, apoE, tau, c  **fos** , neural cell adhesion molecular 180, TGF beta 1, IL 1 alpha beta, IL 6, TNF alpha beta, alpha 2 Macroglobulin, class II major histocompatibility antigen la, bcl 2 alpha, glucocorticoid receptor alpha  [ **NR3C1** ]  and mineralocorticoid receptor mRNAs were determined by reverse transcription polymerase chain reaction in the hippocampus and cerebral cortex of senescence accelerated mouse.  Ref: 10406024 Neurosci Lett, 1999
